# Return to Intended Oncologic Therapy After Brain Metastasis Surgery: Mapping the Early Postoperative Pathway

**DOI:** 10.3390/curroncol33090511

**Published:** 2026-08-27

**Authors:** Alexis Hadjiathanasiou, Martin Liebisch, Johanna Dahn, Johannes Lemcke, Patrick Schuss

**Affiliations:** Department of Neurosurgery, BG Klinikum Unfallkrankenhaus Berlin, 12683 Berlin, Germany

**Keywords:** brain metastases, return to intended oncologic therapy, oncologic readiness

## Abstract

Surgery for brain metastases (BM) is embedded in a multidisciplinary oncologic pathway. This study evaluated oncologic readiness and return to intended oncologic therapy (RIOT) after surgery for BM. Patients undergoing surgery for histologically confirmed BM were retrospectively identified. Oncologic readiness was defined as sufficient postoperative clinical and neurological recovery to allow the next indicated oncologic treatment. RIOT was defined as postoperative radiotherapy and/or systemic treatment within 30 days after surgery. Exploratory analyses assessed factors associated with oncologic readiness and RIOT after readiness. Among 126 patients, oncologic readiness was achieved in 114 (90%) and RIOT in 99 (79%). Absence of oncologic readiness was associated with postoperative morbidity and persistent neurological deficit, whereas absence of RIOT after readiness showed no distinct clinical or BM-related profile. Change in patient preference, neurological or functional deterioration, and organizational delay were the most frequent reasons for no RIOT after readiness. Oncologic readiness distinguished patients with insufficient surgical recovery from those who recovered but did not proceed to further treatment. These findings support RIOT as a process endpoint for the transition from BM surgery to postoperative oncologic care.

## Simple Summary

Surgery for brain metastases is often only one step in a broader cancer treatment plan. After surgery, many patients are expected to continue with radiation therapy or systemic cancer treatment. However, it is not always clear whether treatment is delayed because the patient has not recovered well enough from surgery or because later treatment decisions, patient preferences, or organizational factors have changed the care pathway. In this study of 126 patients, most patients recovered sufficiently to be considered ready for further cancer treatment and most started treatment within 30 days. Patients who did not become ready for treatment more often had postoperative complications or persistent neurological problems. Among patients who were ready but still did not start treatment, the reasons were more varied. These findings may help future studies and clinical teams better understand and improve the transition from brain metastasis surgery to further cancer care.

## 1. Introduction

Brain metastases (BM) are a frequent manifestation of systemic cancer and are associated with substantial morbidity and impaired survival [1]. Local treatment of BM is selected according to the number, size, and location of intracranial lesions, neurological symptoms, performance status, extracranial disease, available systemic treatment options, and overall treatment goals. In patients with localized intracranial metastatic disease, surgery and stereotactic radiosurgery represent established local treatment strategies that require individualized multidisciplinary selection [2]. In selected patients, surgical resection remains an established treatment option to relieve mass effect, improve/stabilize neurological function, obtain histological diagnosis and achieve local tumor control [3]. Surgery may also be considered in patients with multiple BM when a dominant symptomatic or space-occupying lesion requires rapid decompression or when histological confirmation is required [3]. However, surgery for BM is rarely an isolated therapeutic event. It is usually part of a multidisciplinary oncological treatment plan involving postoperative radiation therapy, systemic treatment, a reevaluation of the initial treatment or even best supportive care (BSC) [3,4,5]. Therefore, the value of BM surgery is not only determined by the technical extent and safety of resection, but also by the patient’s ability to recover sufficiently to continue indicated oncologic treatment.

In surgical oncology, return to intended oncologic therapy (RIOT) has been introduced to assess whether patients are able to start the intended postoperative oncologic treatment after cancer surgery [6]. Previous studies in extracranial oncologic surgery have shown that absent RIOT is associated with postoperative complications, prolonged recovery, impaired performance status and adverse oncologic outcome [6,7]. In addition, oncologic (or RIOT) readiness has been proposed to describe the time point at which patients are considered sufficiently recovered to receive further oncologic treatment, irrespective of whether this treatment is ultimately administered [7]. Thus, RIOT and oncologic readiness address related but distinct aspects of the early postoperative course.

Despite the relevance of timely postoperative treatment continuation after BM surgery, RIOT has not been systematically characterized in this patient population. This appears relevant because absence of timely treatment initiation after BM surgery may have different causes. It may reflect insufficient postoperative recovery, but it may also reflect subsequent oncologic decision-making, reassessment of the systemic disease situation, patient preference, treatment redirection or delayed treatment delivery. A binary assessment of RIOT alone may therefore combine clinically different situations into one endpoint. Separating oncologic readiness from RIOT may better identify whether absent timely treatment initiation is related to the neurosurgical recovery phase or to the subsequent oncologic course.

Therefore, in the present study, we aimed to analyze postoperative oncologic readiness and RIOT within 30 days after surgery for BM.

## 2. Materials and Methods

All consecutive adult patients who underwent surgery for brain metastases at the authors’ institution between 2023 and 2025 were identified retrospectively using institutional diagnosis- and procedure-related coding data. Patient characteristics, tumor- and surgery-related variables, postoperative course and oncologic pathway data were extracted and entered into a database. The indication for surgical resection was determined on an individual basis, taking into account neurological symptoms, lesion size and location, mass effect, the need for histological confirmation, extracranial disease status and the anticipated postoperative oncologic treatment strategy. The intended postoperative oncologic treatment was identified from the available preoperative treatment plan and, where this had not yet been finalized, from early postoperative multidisciplinary documentation, including tumor board recommendations and oncologic records. Baseline variables included age, sex, preoperative Karnofsky Performance Status (KPS), Eastern Cooperative Oncology Group (ECOG) performance status, primary tumor site, extracranial disease status, preoperative systemic treatment, neurological symptoms, number and location of brain metastases, midline shift > 5 mm, hydrocephalus and surgery-related variables. Postoperative variables included complications within 30 days, unplanned intensive care unit (ICU)/intermediate care (IMC) readmission, necessity of reoperation, persistent neurological deficit at discharge, discharge destination, oncologic readiness, RIOT and the documented reason for absent RIOT.

Oncologic readiness was defined as sufficient postoperative clinical and neurological recovery to allow initiation of the next indicated oncologic treatment. Readiness required clinical stability, a neurological and functional condition considered sufficient for further oncologic treatment, absence of an unresolved acute postoperative condition precluding treatment and documented eligibility for further oncologic management based on the postoperative clinical course, discharge documentation, oncologic records and/or multidisciplinary recommendations where available. Therefore, oncological readiness was based on the overall postoperative clinical, neurological, and functional course rather than on an isolated performance-status threshold. KPS and ECOG were considered as complementary clinical measures and did not independently determine the readiness classification. Actual treatment initiation was not used to determine readiness. Oncologic readiness was independently assessed by two investigators (AH and ML). Discordant or unclear cases were reviewed by a third investigator (PS) and resolved by consensus.

Return to intended oncologic therapy was defined as initiation of postoperative radiotherapy and/or systemic oncologic treatment within 30 days after surgery for BM [8]. If both treatment modalities were initiated within this interval, the earliest documented start date was used as the RIOT date. Patients without initiation of postoperative radiotherapy or systemic oncologic treatment within 30 days were classified as having no RIOT.

Patients were then divided into three groups based on their postoperative treatment course: oncologic readiness with RIOT, oncologic readiness with no RIOT and no oncologic readiness. For patients with oncologic readiness but no RIOT, the predominant documented reason was classified as patient preference, transition to best supportive care (BSC), neurological or functional deterioration, organizational delay, or medical/surgical complication. When several factors contributed to absent RIOT, the predominant reason was defined as the factor most directly documented as preventing treatment initiation within 30 days, considering the temporal sequence of events and the final documented treatment decision. The classification reflected the dominant documented mechanism and did not exclude additional contributing factors. Ambiguous cases were resolved by consensus.

Data analysis was performed using R (version 4.6.0; R Foundation for Statistical Computing, Vienna, Austria). Categorical variables are presented as numbers and percentages, and continuous variables as median with interquartile range. Unpaired categorical variables were analyzed in contingency tables using Fisher’s exact test. Continuous variables were compared using the Mann–Whitney U test or Kruskal–Wallis test. Exploratory subgroup comparisons were conducted between patients with/without oncologic readiness and between patients with/without RIOT among those with oncologic readiness. Two-sided *p*-values < 0.05 were considered statistically significant. No adjustment for multiple comparisons was performed.

## 3. Results

### 3.1. Patient Characteristics

A total of 126 patients who underwent surgery for BM were included. Median age at surgery was 67 years (IQR 59–74), and 57 patients (45%) were female. Median preoperative KPS was 70 (IQR 60–80), with 36 patients (29%) presenting with a KPS < 70. The most frequent primary tumor site was lung cancer (*n* = 65, 52%), followed by melanoma (*n* = 13, 10%), breast cancer (*n* = 12, 10%), gastrointestinal cancer (*n* = 12, 10%), and other entities (*n* = 24, 19%). Active extracranial disease was documented in 80 patients (63%). Multiple BM were present in 63 patients (50%). Among these patients, 26 had 2–3 BM and 37 presented with >3 BM. The lesion compartment was supratentorial in 99 patients (79%) and infratentorial in 27 patients (21%). Further baseline characteristics are summarized in Table 1.

### 3.2. Postoperative Pathway: Oncologic Readiness and RIOT

**Figure 1:**
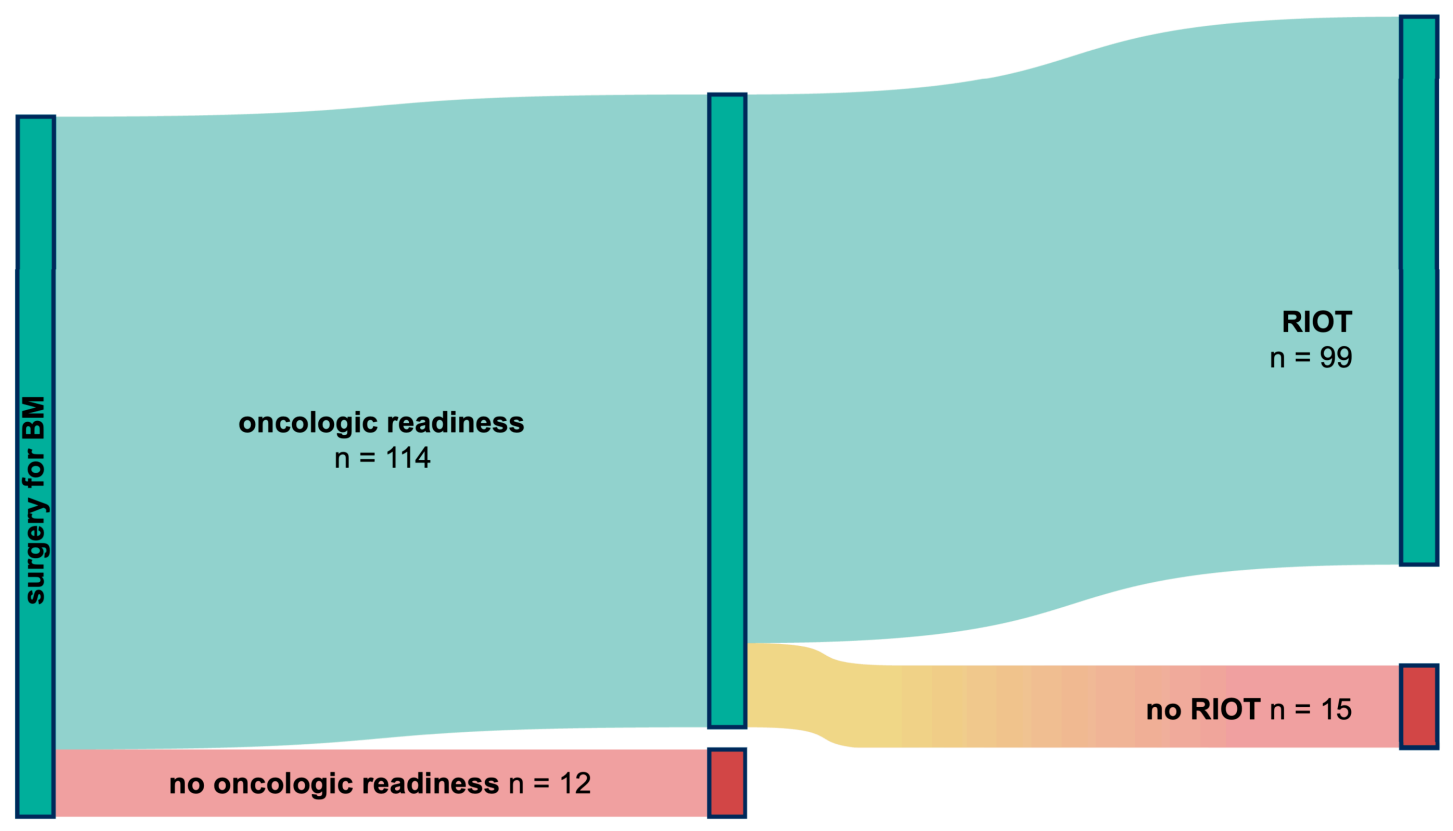
Postoperative pathway from surgery to oncologic readiness and RIOT after surgery for brain metastases. Patients were first classified according to postoperative oncologic readiness. RIOT was defined as initiation of postoperative radiotherapy and/or systemic oncologic treatment within 30 days after surgery. Patients without treatment initiation within this interval were classified as no RIOT.

Postoperative oncologic readiness within 30 days after surgery was documented in 114 of 126 patients (90%), whereas 12 patients (10%) did not achieve oncologic readiness. Among the 114 patients with oncologic readiness, 99 patients (87%) underwent RIOT, whereas 15 patients (13%) received no RIOT. Overall, RIOT was achieved in 99 of 126 patients (79%), whereas 27 patients (21%) had no RIOT. The early postoperative pathway according to oncologic readiness and subsequent RIOT status is shown in Figure 1.

### 3.3. Reasons for No RIOT After Oncologic Readiness

**Figure 2:**
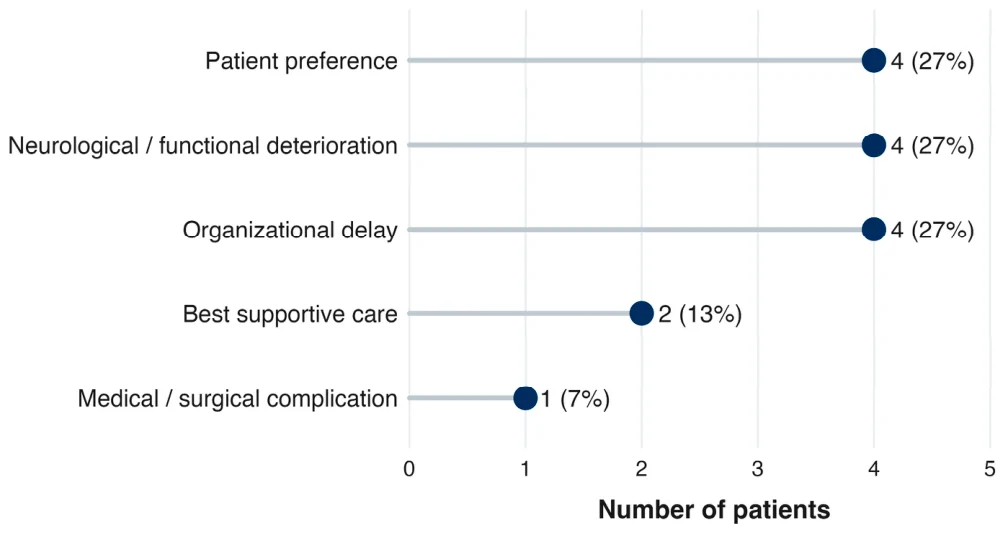
Reasons for no RIOT after oncologic readiness. Reasons are shown for patients who achieved postoperative oncologic readiness but did not initiate postoperative radiotherapy or systemic oncologic treatment within 30 days after surgery.

The group with absent RIOT consisted of 15 patients with and 12 patients without previous postoperative oncologic readiness. Among those 15 patients with oncologic readiness but without subsequent RIOT, change in patient preference, secondary neurological/functional deterioration and organizational delays were the predominant reasons. Among the 15 patients with oncologic readiness but no RIOT, three required unplanned readmission within 30 days after initial discharge home. The reasons were a new epileptic seizure, functional deterioration associated with urosepsis and a fall resulting in a traumatic subdural hematoma requiring evacuation. In all three patients, the intervening event prevented RIOT within the predefined 30-day interval. The distribution of reasons for the absence of RIOT following oncologic readiness is presented in Figure 2.

### 3.4. Clinical Factors Associated with Oncologic Readiness

In exploratory subgroup comparisons, patients without oncologic readiness showed a distinct perioperative profile compared with patients with oncologic readiness. Preoperative KPS ≥ 70 was less frequent in patients without oncologic readiness (42% vs. 75%, *p* = 0.038), whereas hydrocephalus was more common (33% vs. 5%, *p* = 0.007). Furthermore, patients without oncologic readiness more frequently experienced any 30-day complication (67% vs. 20%, *p* = 0.001), unplanned ICU/IMC readmission (33% vs. 2%, *p* < 0.001), reoperation within 30 days (25% vs. 1%, *p* = 0.003) and persistent neurological deficit at discharge (67% vs. 18%, *p* < 0.001). These findings are summarized in Table 2.

### 3.5. RIOT Status Among Patients with Oncologic Readiness

In exploratory subgroup comparisons among patients with oncologic readiness, failure of RIOT was not associated with specific features regarding the baseline situation, characteristics/effects of the space-occupying lesions, or perioperative morbidity. However, the necessity of an unplanned readmission within 30 days occurred more frequently in patients with oncologic readiness who did not achieve RIOT than in patients with oncologic readiness and subsequent RIOT (20% vs. 3%, *p* = 0.029). Among the three readmitted patients who nevertheless achieved RIOT, two were readmitted for secondary pulmonary infections requiring antibiotic treatment and one after a fall with head impact requiring evaluation for traumatic brain injury. All three patients with unplanned readmission subsequently initiated oncologic treatment within 30 days. The detailed results are summarized in Table 3.

## 4. Discussion

The present study investigated oncologic readiness and return to intended oncologic therapy after surgery for brain metastases. RIOT was introduced as a surgical oncology metric to assess whether patients are able to start the intended postoperative oncologic treatment after cancer surgery [6,7]. In the present cohort, oncologic readiness was achieved in 90% of patients, whereas RIOT within 30 days was achieved in 79% of patients after BM surgery. Thus, most patients recovered sufficiently to allow further oncologic treatment, but timely treatment initiation was not achieved in all patients within the predefined postoperative interval. The relatively high proportion of patients with multiple BM should be interpreted in the context of this surgically selected cohort. In these patients, the indication for resection was based on the clinical relevance of the surgical target, including neurological symptoms, lesion size and location, mass effect, the need for histological confirmation and the anticipated postoperative oncologic strategy, rather than on intracranial lesion number alone.

The distinction between oncologic readiness and RIOT is particularly relevant in BM surgery. In contrast to many extracranial oncologic procedures, surgery for BM is commonly followed by treatment decisions and treatment delivery across different disciplines, including neurosurgery, radiation oncology and medical oncology [3,4,5]. Accordingly, the absence of RIOT may have heterogeneous causes. In addition to inadequate postoperative recovery or a decline in neurological/physical reserves, other potential causes for the absence of RIOT include factors related to subsequent oncological decision-making, obstacles in treatment logistics and/or changes in or consideration of the patient’s preferences, including the initiation of BSC. By covering the recovery-related component of absent subsequent treatment, oncologic readiness constitutes a postoperative intermediate endpoint. As it precedes the actual start of treatment, oncologic readiness might provide insight into the interpretative significance or cause of the absence of RIOT following BM surgery.

Patients without oncologic readiness predominantly represented the perioperative part of absent RIOT. In the present study, absence of oncologic readiness was associated with lower preoperative KPS, preoperative hydrocephalus, postoperative complications, unplanned ICU/IMC readmission, reoperation, as well as persistent neurological deficit at discharge. This profile is in line with previous data indicating that functional status after BM surgery is closely related to the ability to receive subsequent oncologic treatment [9]. In addition, postoperative adverse events and prolonged postoperative intensive care have previously been identified as relevant factors after surgical treatment of BM and have been associated with impaired survival [10,11]. Thus, patients without oncologic readiness seem to represent a clinically visible subgroup with limited neurological reserve and an unfavorable early postoperative course. In this subgroup, failure to initiate postoperative oncologic treatment might primarily pose a problem in terms of recovery. Therefore, careful patient selection, preservation of neurological function and avoidance of postoperative morbidity remain essential when BM surgery is intended to enable further oncologic treatment [12,13].

A different finding was observed in patients who achieved oncologic readiness but did not undergo RIOT. In this subgroup, absence of RIOT was not associated with a distinct baseline, BM-related or perioperative variables. This finding suggests that absent RIOT after initial readiness should not be interpreted as residual perioperative morbidity alone. In previous extracranial RIOT studies, failure to return to postoperative oncologic treatment was closely linked to postoperative recovery and complications [6,7]. In the present BM cohort, this association was mainly observed in patients who did not achieve oncologic readiness. Once oncologic readiness had been achieved, absence of RIOT was not associated with a distinct pattern of the recorded baseline or perioperative variables. However, unplanned readmission within 30 days was more frequent among patients with oncologic readiness but no RIOT, indicating that clinical vulnerability may persist or recur after initial recovery and may still interfere with treatment initiation. Other documented reasons for the absence of RIOT after initial oncological readiness included the oncological disease situation, patient preference, treatment logistics and reassessment of treatment goals. This interpretation is consistent with current BM recommendations, which emphasize that postoperative treatment decisions should be based on multidisciplinary assessment of histology, neurological status, extracranial disease, available treatment options, prognosis and goals of care [3,4]. In this context, absence of RIOT after previous oncologic readiness may reflect a revised oncologic indication, a change in patient preference or the time needed for tumor board review and communication of treatment goals. These factors are heterogeneous and highly individual. Nevertheless, parts of this transition may be amenable to more structured interdisciplinary communication, earlier clarification of the responsible treating discipline and support during shared decision-making, rather than to further perioperative risk stratification alone [14,15].

Several limitations should be considered. This was a retrospective single-center study, and oncologic readiness was assessed from the documented postoperative course rather than by a prospectively defined score. As a single-center surgical cohort, the study may be subject to referral and selection bias and may not be generalizable to the broader population of patients with BM. In addition, the study was designed around the predefined binary 30-day RIOT endpoint, whereas exact day-level treatment intervals were not consistently available with sufficient completeness for a robust time-to-RIOT analysis. The reason for absent RIOT was classified according to the dominant documented mechanism, although more than one factor may have contributed in individual patients. Subgroup analyses were limited by the small number of patients in the respective groups and should therefore be interpreted as exploratory. No adjustment for multiple comparisons was performed. The study was designed to characterize the early postoperative pathway from surgery to oncologic readiness and treatment initiation, whereas the subsequent treatment period beyond the immediate neurosurgical transition was outside the scope of the present analysis.

To our knowledge, the present study is the first to apply RIOT to the early postoperative course after surgery for brain metastases. Its main contribution is the separation of two situations that a binary RIOT endpoint combines into one. In patients who did not reach oncologic readiness, the obstacle to further treatment was surgical recovery. In patients who reached readiness but did not undergo RIOT, the absence of treatment reflected the subsequent oncologic course, clinical changes, patient preference or organizational delay rather than surgical recovery. Without the readiness step, both groups are counted together as no RIOT. Neither endpoint was intended to measure survival, intracranial tumor control, or the quality of subsequent radiotherapy/systemic treatment or multidisciplinary care. Both describe only the early postoperative transition from surgery to further oncologic treatment, and oncologic readiness identifies where in that transition the absence of RIOT arises.

## 5. Conclusions

RIOT provides a clinically applicable postoperative endpoint for whether intended oncologic treatment is initiated after surgery for BM. Oncologic readiness may add an interpretive step by identifying whether a patient has initially recovered sufficiently to proceed to further treatment. In this cohort, failure to achieve readiness was associated with postoperative morbidity and persistent neurological impairment, whereas no RIOT after readiness was not characterized by a comparable perioperative profile and reflected a more heterogeneous subsequent course. Later clinical events could nevertheless still interfere with treatment initiation. However, prospective validation is required before oncologic readiness is established as a standardized companion measure to RIOT.

## Figures and Tables

**Table 1:** Baseline characteristics of the study cohort.

Characteristic	Patients (*n* = 126)
Median age at surgery (yrs)	67 (IQR 59–74)
Female sex	57 (45%)
Preoperative KPS < 70	36 (29%)
Preoperative ECOG ≥ 3	46 (37%)
Known cancer before BM surgery	63 (50%)
Systemic treatment ≤ 30 days preoperatively	11 (9%)
Preoperative neurological deficit	58 (46%)
Preoperative seizure	27 (21%)
Primary tumor site	
Lung	65 (52%)
Melanoma	13 (10%)
Breast	12 (10%)
Gastrointestinal	12 (10%)
Other	24 (19%)
Multiple BM	63 (50%)
Infratentorial BM	27 (21%)
Midline shift > 5 mm	21 (17%)
Hydrocephalus	10 (8%)
Multiple BM resected during index surgery	10 (8%)
Intraoperative complication	3 (2%)

**Table 2:** Factors associated with postoperative oncologic readiness.

Characteristic	Postoperative Oncologic Readiness	No Oncologic Readiness	*p*-Value
Age ≥ 70	45 (39%)	5 (42%)	1.000
Preoperative KPS ≥ 70	85 (75%)	5 (42%)	0.038
Known cancer before BM surgery	54 (47%)	9 (75%)	0.126
Multiple BM	58 (51%)	5 (42%)	0.763
Infratentorial BM	22 (19%)	5 (42%)	0.130
Preoperative hydrocephalus	6 (5%)	4 (33%)	0.007
Any postoperative complication	23 (20%)	8 (67%)	0.001
Unplanned ICU/IMC readmission	2 (2%)	4 (33%)	<0.001
Reoperation within 30 days	1 (1%)	3 (25%)	0.003
Persistent neurological deficit at discharge	20 (18%)	8 (67%)	<0.001

**Table 3:** Factors associated with RIOT after postoperative oncologic readiness.

Characteristic	Oncologic Readiness with RIOT	Oncologic Readiness Without RIOT	*p*-Value
Age ≥ 70	40 (40%)	5 (33%)	0.779
Preoperative KPS ≥ 70	74 (75%)	11 (73%)	1.000
Known cancer before BM surgery	46 (46%)	8 (53%)	0.783
Multiple BM	47 (47%)	11 (73%)	0.095
Infratentorial BM	21 (21%)	1 (7%)	0.296
Preoperative hydrocephalus	6 (6%)	0 (0%)	1.000
Any postoperative complication	19 (19%)	4 (27%)	0.499
Unplanned ICU/IMC readmission	2 (2%)	0 (0%)	1.000
Reoperation within 30 days	1 (1%)	0 (0%)	1.000
Persistent neurological deficit at discharge	19 (19%)	1 (7%)	0.464
Unplanned readmission within 30 days	3 (3%)	3 (20%)	0.029

## Data Availability

The original contributions presented in this study are included in the article. Further inquiries can be directed to the corresponding author.

## Abbreviations

The following abbreviations are used in this manuscript:

BM	Brain metastases
BSC	Best supportive care
ECOG	Eastern Cooperative Oncology Group
ICU	Intensive care unit
IMC	Intermediate care
IQR	Interquartile range
KPS	Karnofsky Performance Status
RIOT	Return to intended oncologic therapy
yrs	years
